# ABO and RhD blood groups and their relationship with diseases: A retrospective study

**DOI:** 10.12669/pjms.40.4.8663

**Published:** 2024

**Authors:** Olgun Goktas

**Affiliations:** 1 Dr. Olgun Goktas Associate Professor, Uludag University Family Health Center, Nilufer, Bursa, Turkey

**Keywords:** ABO, Blood groups, Diseases, Disorders, Rhesus factor

## Abstract

**Objective:**

To retrospectively evaluate the distribution of ABO and RhD blood groups and their relationship with diseases.

**Methods:**

The retrospective study was carried out in Bursa Uludag University Family Health Center in Turkey between 1-28 February, 2023. The data of individuals who were registered with the Family Health Center and whose blood types were known were evaluated retrospectively. Blood group type, sociodemographic findings, existing diseases, allergies, and genetic disease conditions were obtained. *P-values* below 0.05 were considered statistically significant. Analysis were made in the SPSS 25.0 program.

**Results:**

A total of 3834 people, 1935 male (50.5%) and 1899 female (49.5%) participated in the study. The mean age of the individuals was 26.45±10.45 years. About 75.8 percent of the participants were university students, 6.3 percent were health workers, and 17.9 percent were from other occupational groups. The rates of blood groups were determined as O Rh D+ at 33.5%, AB Rh D+ at 26.9%, A Rh D+ at 14.9%, AB Rh D- at 7.7%, B Rh D+ at 7%, O Rh D- at 4.6%, B Rh D- at 3.2% and A Rh D- at 2.2%. It was determined that the O Rh D- group had a higher rate of genetic disease than the other groups (p=0.01).

**Conclusion:**

It was determined that the O Rh D+ blood group was found more frequently in our region’s population than in other groups. This different result depending on the A Rh D+ blood group, which is the most common throughout the country may have reflected the influence of different geographical regions and ethnic characteristics due to the students in our population. In addition, the results regarding the relationships between blood groups and occupation, genetics, existing disease, presence of allergies, and visual defects in the eye are important.

## INTRODUCTION

It was known about a century ago that the observed distribution of human blood groups differs according to countries and regions. It is debated whether the differences are the result of genetic factors and constitutive effects in small populations, or whether they are the result of natural selection resulting from differences in fitness between groups.

The differences are also thought to be due to the locally determined characteristics of the external environment.^1^ These differences are thought to be affected by both conditions, but their relative implications have not yet been clarified.^2^ Although it has been stated that blood groups may be associated with some diseases, especially those connected to the ABO system, new studies are needed. There are insufficient studies with large samples in the literature on which diseases ABO and RhD blood groups may be increasingly associated .^3^

The most current analysis **of** the distribution of blood groups in Turkey is a study conducted in Istanbul. Although it does not reflect the whole country, it gives an idea about the distribution due to its large population. It is noteworthy that the differences between regions have been determined. There is no study on a large scale in the countries of the world and the information is insufficient.^4^,^5^

It emphasizes that genomics is important in all fields of medicine, and that whole genome sequencing, which will be included in the medical records of patients in terms of general health and chronic disease, will provide a suitable blood group profile, especially in terms of blood transfusions.^6^

In a study emphasizing the importance of blood groups in terms of infectious diseases, it is emphasized that the immune response of individuals to infections may vary according to blood groups. In this study, it is stated that microorganisms can stimulate antibodies against blood group antigens.^7^ A, B, O, and AB blood groups and Rh positive and Rh negative status are important in genetics.^8^ It has been reported that some infectious diseases are seen especially in certain blood groups.^9^

In the literature, information on the distribution of blood groups both in Turkey and in the world is based on ancient times and is insufficient. There is not much large-scale data obtained from general studies, especially on diseases and conditions that may affect health. This creates a gap in the literature. Evaluation of registered individual data in family medicine is important in this respect.

In this study, we aimed to investigate the distribution of the ratios of these basic blood groups and their relationship between disease and health status in our individuals registered at a family health center.

## METHODS

This retrospective study was carried out at Bursa Uludağ University Family Health Center between 1-28 February 2023. The data of individuals registered in the Family Health Center and whose blood type (determined by the forward and reverse technique in the ABO system, and the Anti-D reagent for Rh D antigen) is known were evaluated retrospectively. Individual data such as blood group type, age, gender, educational status, previous surgery history, any disease, allergy, visual impairment, genetic disease, and any disease during the research period were obtained from the family medicine information system anonymously.

### Ethical approval

The study was performed after approval from the Clinical Research Ethics Committee of Bursa Uludağ University, Faculty of Medicine (Ref. dated 2023-03-07/ decision no:2023-5/14) following the Declaration of Helsinki.

### Statistical analysis

In this study, the number of observations (n), mean , and standard deviation (s.s.) for the clinical and demographic variables of the individuals are given. The chi-square test was used for the proportional comparison of gender, occupation, genetic disease, current disease, age group, allergy status, visual impairment and history of surgery, and blood groups. Bonferroni test was used to determine the groups that made the difference in the groups that were different. Analysis of variance was performed in the analysis of age measurements according to blood groups. Sidak pairwise comparison test was applied to determine the group that caused the difference in the groups. *P-values* less than 0.05 were considered statistically significant in the study. Analyzes were made with the SPSS 25.0 package program.

## RESULTS

A total of 3834 people, 1935 male (50.5%) and 1899 female (49.5%) participated in the study. The mean age of the individuals was 26.45±10.45 years. Participants were 75.8% university students, 6.3% health workers, and 17.9% from other occupational groups. The rates of blood groups were determined as O Rh D+ at 33.5%, AB Rh D+ at 26.9%, A Rh D+ at 14.9%, AB Rh D- at 7.7%, B Rh D+ at 7%, O Rh D- at 4.6%, B Rh D- at 3.2% and A Rh D- at 2.2%. In addition, it was determined whether the individuals had a genetic disease, any existing disease, allergies, visual defects in the eye, and a history of surgery, (Table-I).

**Table-I:** Demographic data and characteristics of individuals.

		n	%
Gender	Male	1935	50,5%
	Female	1899	49,5%
Occupation	Student	2905	75,8%
	Health employee	243	6,3%
	Other professions	686	17,9%
Blood group	O Rh D+	1284	33,5%
	AB Rh D+	1033	26,9%
	A Rh D+	572	14,9%
	AB Rh D-	294	7,7%
	B Rh D+	269	7,0%
	O Rh D-	176	4,6%
	B Rh D-	123	3,2%
	A Rh D-	83	2,2%
Genetic disease	No	3732	97,3%
	Yes	102	2,7%
Current disease	No	2829	73,8%
	Yes	1005	26,2%
Allergy	No	3636	94,8%
	Yes	198	5,2%
Visual impairments	No	2956	77,1%
	Yes	878	22,9%
Surgical history	No	3528	92,0%
	Yes	306	8,0%
Age group	18 and Under	242	6,3%
	19-40	3213	83,8%
	41 and Above	379	9,9%

It was determined that the genders of the individuals did not differ according to their blood groups. The ratio of women and men did not differ between O Rh D+, O Rh D-, A Rh D+, A Rh D-, B Rh D+, B Rh D-, AB Rh D+, and AB Rh D- groups (p=0.21, p>0.05).Occupations of the individuals differed according to their blood groups. In the study, it was determined that the O Rh D+, AB Rh D+, and AB Rh D- groups were higher in students, and the A Rh D- group was higher in healthcare workers. The B Rh D- group was found to be less common than other occupational groups (p=0.01).

The blood groups of the individuals differed according to the age groups. In the study, it was determined that O Rh D+, O Rh D-, A Rh D+, A Rh D-, B Rh D+, B Rh D-, AB Rh D+, and AB Rh D- groups had similar rates in patients under 18, 19-40 and over 40 years old (p=0.14,). On the other hand, it was observed that the average age of the individuals did not differ according to their blood groups. O Rh D+ and AB Rh D+ groups were found to be younger than B Rh D+ and AB Rh D- groups (p=0.01).

Having a genetic disease, having the current disease, and being allergic differed according to the blood groups of the individuals. Accordingly, the O Rh D- group was found to have a higher genetic disease than the other groups (p=0.01), and the A Rh D- group had a lower rate of any existing disease than the other groups (p=0.01). In addition to these, a higher rate of allergy was observed in O Rh D+, O Rh D-, A Rh D+, and B Rh D+ groups compared to other groups (p=0.04).

Visual impairment in the eyes of the individuals did not differ according to their blood groups, and the B Rh D- groups had a lower rate of visual impairment in the eyes than the other groups (p=0.01). The history of surgery did not differ according to the blood groups of the individuals, and O Rh D+, O Rh D-, A Rh D+, A Rh D-, B Rh D+, B Rh D-, AB Rh D+, AB Rh D- groups were found to have similar rates in patients with or without a history (p=0.12), (Table II).

**Table-II:** Examination of individual characteristics according to blood group:

		Blood Group																p

		O Rh D+		O Rh D-		A Rh D+		A Rh D-		B Rh D+		B Rh D-		AB Rh D+		AB Rh D-		

		n	%	n	%	n	%	n	%	n	%	n	%	n	%	n	%	
Gender	Male	655	51.0	96	54.5	286	50.0	49	59.0	141	52.4	57	46,3	513	49,7	138	46.9	0.21
	Female	629	49.0	80	45.5	286	50.0	34	41.0	128	47.6	66	53,7	520	50,3	156	53.1	
Occupation	Student	1010	78.7	112	63.6	400	69.9	50	60.2	193	71.7	109	88,6	808	78,2	223	75.9	0.01*
	Health employee	76	5.9	15	8.5	45	7.9	13	15.7	13	4,8	5	4,1	56	5,4	20	6.8	
	Other professions	198	15.4	49	27.8	127	22.2	20	24.1	63	23.4	9	7,3	169	16,4	51	17.3	
Age group	18 and Under	105	8.2	20	11,4	33	5.8	7	8,4	11	4.1	2	1,6	57	5,5	7	2.4	0.14
	19-40	1075	83.7	132	75,0	468	81,8	71	85.5	222	82.5	112	91,1	883	85,5	250	85.0	
	41 and Above	104	8.1	24	13.6	71	12.4	5	6.0	36	13.4	9	7,3	93	9,0	37	12.6	
Genetic disease	No	1251	97,4	152	86.4	558	97.6	80	96.4	261	97.0	120	97,6	1023	99,0	287	97.6	0.01*
	Yes	33	2,6	24	13.6	14	2.4	3	3.6	8	3.0	3	2,4	10	1,0	7	2.4	
Current disease	No	922	71.8	121	68.8	397	69,4	71	85.5	191	71,0	110	89,4	805	77,9	212	72.1	0.01*
	Yes	362	28.2	55	31,3	175	30,6	12	14.5	78	29.0	13	10,6	228	22,1	82	27.9	
Allergy	No	1178	91.7	159	90.3	541	94,6	81	97.6	258	95.9	122	99,2	1010	97,8	287	97.6	0.04*
	Yes	106	8.3	17	9.7	31	5,4	2	2,4	11	4.1	1	0,8	23	2,2	7	2.4	
Visual impairments	No	974	75.9	142	80.7	431	75.3	68	81.9	202	75,1	114	92,7	808	78,2	217	73.8	0.01*
	Yes	310	24.1	34	19.3	141	24.7	15	18.1	67	24,9	9	7,3	225	21,8	77	26.2	
Surgical history	No	1189	92.6	162	92.0	512	89.5	79	95.2	245	91.1	120	97,6	952	92,2	269	91.5	0.12
	Yes	95	7.4	14	8.0	60	10.5	4	4.8	24	8.9	3	2,4	81	7,8	25	8.5	
Age group	X+s.s.	25.62	9.30	27.05	11.19	27.41	11.57	26.81	8.72	28.62	11.31	26.18	7,47	25,83	9,66	28.21	11.01	0.01*

* Significant difference at 0.05 level.

## DISCUSSION

This study showed that the O Rh D+ blood group was found more frequently in our region’s population than in other groups. This result was different from the higher frequency of the A Rh D+ group across the country. This difference may be due to different ethnic students coming from abroad.

In addition to the lack of current studies in Turkey, most studies and a study conducted in Istanbul found that the A Rh D+ blood group was more common. On the other hand, the most common blood group in our study was O Rh D+. Our study is region-based and this different outcome is likely due to the inclusion of foreign students of different ethnic origins coming from abroad for study. Similar to our study, O Rh D+ was found more frequently in a study in a university student population in Somalia.^10^

A study conducted in 67 different populations from different countries of the world, it was shown that the interaction between ABO and Rh blood groups creates the selection between genes controlling the A2 antigen (p2) and the Rh-negative phenotype (r).^11^ A population-based study conducted in China showed that the ABO and RhD phenotypes differed significantly in nine different ethnic groups.^12^

In a study in England, it was determined that Group-B parents gave birth to more boys in heterozygous matings and at first birth.^13^ In the study conducted by Sanhgvi, the percentage of boys was higher in Group-O babies of Group-O mothers compared to Group-A babies of Group-A mothers.^14^ In our study, the sex of the individuals according to their blood groups did not differ in all blood groups. While no gender difference was found in a study between student groups, it was determined that Group-B was more common in students from one region, and Group-O in students from another region.^15^

Relationships between blood type, and disease and genetic characteristics are important. In another study it was emphasized that antigens of blood groups are secondary genes because antigens and antibodies are hereditary, and essential enzymes are primary genes.^16^ It is recommended to add a comprehensive antibody screening to antenatal care for hemolytic disease of the newborn against a possible risk of birth of blood groups.^17^ Similarly, it has been reported that obesity is more common in O Rh D+ blood groups.^18^ It has been shown that individuals with Group-A develop ischemic heart disease at a higher rate than other blood groups.^19^-^21^ It is emphasized that some enzyme deficiencies in humans form the basis of blood groups and this situation affects the risk of infection, infectious disease, and blood and organ transplantation.^22^

In our study, it was recorded whether the individuals had any existing disease or genetic disease during the study period. We observed that the prevalence of having any existing disease and genetic disease differ according to the blood groups of the individuals. Accordingly, it was determined that the A Rh D- group had any disease at a lower rate than the other groups. In addition, it was noted that the O Rh D- group had a higher rate of genetic disease than other blood groups. We also found that the B Rh D- blood group had visual defects at a lower rate than the other groups.

In another study, blood Group-B had the highest frequency of secretion, while blood Group-AB had the lowest frequency of secretion.^23^ In another study, the relationship of blood groups with disease and age was reviewed. In this study, the effect of the association of blood group antigens with the interleukin (IL)-6 gene in the inflammatory process on cardiovascular disease formation and longevity is explained.^24^ In our study, when the average age groups were taken, it was determined that the O Rh D+ and AB Rh D+ groups were younger than the B Rh D+ and AB Rh D- groups.

In a study conducted on ABO blood groups, it was determined that allergic rhinitis and asthma were more common in the O blood group, whereas atopic dermatitis was seen more frequently in A and B groups.^25^ It was shown that allergic rhinitis was more common in males in the O blood group phenotype.^26^ We determined that the rate of allergy according to blood groups of individuals differed. Accordingly, we found that the group of O Rh D+, O Rh D-, A Rh D+, and B Rh D+ groups had a higher rate of allergy than the other groups.

As a result of our study, the fact that the most common blood group is different from the countrywide indicates that the factors affecting this situation should be investigated. In addition, the fact that some blood groups are seen less and more frequently in some diseases and disorders is important to consider some factors in clinical practice.

### Limitations

In our study, there was no laboratory data on subtypes of ABO blood groups (eg, A1, A2, A3, Aw, Ax, and Ael), so no evaluation could be made.

## CONCLUSION

We found that the O Rh D+ blood group were more frequently prevalent in our region’s population than in other groups. This result was different from the higher frequency of the A Rh D+ group across the country. This different result may be due to different ethnic students coming from abroad. In this study, we also found that the A Rh D- group had a lower prevalence of any existing disease than other groups, and the O Rh D- group had a higher rate of having any genetic disease compared to other blood groups. Visual impairment in the B Rh D+ blood group was less than in the other groups. We also observed that allergic diseases were more common in O Rh D+, O Rh D-, A Rh D+, and B Rh D+ blood groups compared to other groups. Our results regarding the relationship between blood groups and diseases of individuals in our region’s population will contribute to new studies in the future.
